# The Boot on the Other Leg

**Published:** 1858-01

**Authors:** 


*The Boot on the other Leg.*—In the July number of the *North American Medico-Chirurgical Review* is an article written to prove that an English writer on the microscope, Mr. Hogg, had deliberately appropriated a part of a work on the same subject, by Dr. Wythe, without making the slightest acknowledgment. A letter from Mr. Hogg to the editor of the London *Medical Circular* places the matter in a different light, by showing that Dr. Wythe had copied largely from the "Practical Treatise on the Microscope," by Prof. Quekett, and from a work by Miss Agnes Catlow, with the title of "Drops of Water," not only whole pages of text, but also wood cuts and plates. These proceedings of Dr. Wythe are severely animadverted upon by the editor of the *Quarterly Journal of Microscopical Science*, vol. i., 1853, who says, "on account of the proved plagiarism of this part of the work (the description of the infusoria,) we understand the publishers of Miss Catlow's work have been enabled to prevent the further sale of the American work." Mr. Hogg says, in conclusion, "The apparent resemblance in some of the American author's productions and my own, is therefore readily explained and easily understood, when it is known that I drew upon our English authors with *their knowledge, and in most instances with their consent*, which was invariably granted." We print the above in justice to Mr. Hogg, having in a former number called attention to the accusation in the *North American Medico-Chirurgical Review.—Boston Medical and Surgical Journal.*

